# Cases from the Note Book of Robert Robson, M. D., with Observations

**Published:** 1872-02-01

**Authors:** 


### Original Communications.

### CASES FROM THE NOTE BOOK OF ROBERT ROBSON, M. D., WITH OBSERVATIONS.

CASE I.—DISEASE OF THE FUNIS UMBILICALIS.

A few days ago I was called to attend a lady in labor with her second child, living four miles from my residence. On my arrival I was told it was all over, the child born and the placenta expelled, and the lady all right, but that there was something serious the matter with the child which they had never seen before, and on inspection of the child, to my surprise, there were three distinct tumors resting on the abdomen, and connected with the umbilicus, close to the integument of the navel. The one containing the cord was about the length and circumference of an ounce quinine jar, with a continuation of a small, shriveled cord, projecting from its extremity. The under part of this tumor was of firm, compact, tissue, while the upper, or superior portion of it, was soft and elastic, without any pulsation, and of a dirty, livid color. Immediately beneath, growing from the above, at its junction with the abdomen, was another of different character, consisting of a transparent globular mass, of the size of a large orange, and another of the size of a pullet's egg, containing a thick albuminous substance, like jelly, both of which I extirpated. The following day I returned, and cut loose from the abdomen of the child this disgusting looking tumor, leaving a small button of flesh, in order to prevent the ligature from slipping off at the navel. The amount of blood lost was not less than three or four ounces, which materially diminished the bulk of the tumor. I carried the tumor home with me, and in company with Dr. Rollins, of this place, made an examination of its contents, which consisted mainly of the funis in a spiral form, each coil adhering to the other, and thoroughly agglutinated with albuminous substances, some extravasated blood, and with here and there some points of organized matter.

The child was lean and feeble, weighing about five pounds, imperfectly developed in the lower extremities, the limbs being very small, and consisting alone of bone and integument, while its head, trunk and superior extremities, were very little better.

*Observations.*—On inquiry of the woman in reference to her health during the period of gestation, she observed it had been good during the entire period, with only one exception, that some months previous to the birth of her child she had come in contact with a large snake, and on the day following this circumstance, with another, which alarmed her very much. Whatever effect the fright may have had in this case on the gravid uterus and appendages through the nervous system, I am not prepared to offer any conjectures. That the child was lean, feeble, and imperfectly developed, was undoubtedly the result of an arrest of growth in consequence of diminished nutrition.

It is very difficult to understand how the fœtal circulation could perform its function, through such a mass of matter, as the above disease of the funis presented. I regret I did not have an opportunity of seeing the placenta. The child died on the third day after birth.

CASE II.—SPONTANEOUS OCCLUSION OF THE URETHRA OF THE PENIS.

A gentleman, advanced in years, the father of a large family, called at my office, and stated that he had experienced much difficulty in urinating for the last two or three years, and that he now feared the canal through the penis was completely obliterated, that his brother was in a similar condition, and that his father, during the latter part of his life, was subject to the same difficulty.

On examination I found the bladder painfully distended, but failed to perceive the least sign of a urethra, and it was only after using a magnifying glass that I could discover a mere speck, representing the urethra. I attempted to introduce the smallest size probe, but without success, and again penetrated the penis some distance further, which proved sufficient. He was now able to urinate. The loss of blood from so small an operation was considerable. The remaining treatment consisted in dilating the urethra by injections of tepid water, and smearing the point of the syringe at every attempt with the cerat. simplex, to prevent cohesion. After continuing this course for two or three days he called again to say he had no further difficulty.

*Observations.*—This is the second case of this character ocurring in old subjects, which has come under my notice lately. From the history of the above case, there would appear to be a hereditary tendency in the family to lesions of nutrition in the mucous membrane of the urethra, in consequence, I suppose, of some modification of the capillary circulation of the part. I have seen various degrees of narrowing in the urethra of boys, and the same cause which produces it in the boy will undoubtedly produce it in the adult. Cases present themselves of chronic inflammation, from gonorrhea, of the corpus spongiosum urethra, but in such cases the spongiosum may be felt indurated with more or less discharge from the urethra, but in the above instance nothing of the kind has ever been the case with my patient. That there must have been chronic inflammation there can be no doubt, but from what source?

### CASE III.—HYDRATE OF CHLORAL IN ASTHMA.

Asthma is often so obstinate and unmanageable, and so difficult to treat successfully, that any remedial agent that promises to be of value, is important, and deserves a trial at the hands of the profession; and it may therefore be not altogether unprofitable on my part to report the following case: A lady of about thirty-six years of age, of rather delicate constitution, subject to asthma, was seized with a violent paroxysm of spasmodic asthma, and sent for her physician, who was not at home, when I was called in, at six o'clock in the evening, to attend her. She had then been suffering twelve hours. I found her, on my arrival, laboring hard from want of breath, almost suffocating, and a loud, wheezing noise, and rattling, from effusion of mucus in the pulmonary tissues. I administered a mixture of Hoff's anodyne, compound syrup of squills, and fluid extract valerian, in large teaspoonful doses from time to time, when the squills caused her to throw up a quantity of mucus, which gave great relief at the moment, and induced a sleep of half an hour. On waking up, the paroxysm returned, with an aggravation of all the symptoms, and with great oppression and dyspnœa; she desired the doors to be thrown open—more air was the cry. At this moment her pulse was small and weak, her skin cold, her countenance indicative of great suffering. I now added some hyosciamus to my prescription, but nothing I had done, or could suggest, did any good. She seemed to be getting worse, and I knew of no practice which experience has proved generally successful, or of no pathological light to direct me to the radical cure or relief of this most distressing disease. Antispasmodics, anodynes, expectorants, and the datura stramonium, which was repeatedly smoked during the intervals of the paroxysms, all failing, which had given great relief in her previous attacks.

Entertaining, among the various pathological views of this disease, the impression that the disease was one of spasmodic action of the excito-motory nerves, and most probably consequent on visceral derangement, I resolved to change my treatment, my first indication being to relieve the pain and dyspnœa, and induce sleep. For this purpose I used the following prescription: ℞ Hydrate of chloral ℈ij, acet. scillæ, tinct. opii camph. aa ℥ss, one-fourth of which was given every hour. After the second dose, the pains and dyspnœa subsided, and she fell into a sound uninterrupted sleep, which continued for some hours, and awoke comparatively well, expectorates freely, and has been improving without any further treatment.

